# 

**Published:** 1905-08-12

**Authors:** 


NOTICE TO CORRESPONDENTS.

All MSS., Letters, Books for Review, and other matters intended for the Editor should be addressed The Editor, "The Hospital," The Hospital Buildings, 28 & 29 Southampton Street, Strand, W.C.

The Editor cannot undertake to return rejected MS., even when accompanied by stamped directed envelope.

**Notice to Advertisers and Subscribers.**

All Advertisements, Orders for Copies of The Hospital, and Business Communications should be addressed to **THE MANAGER (Not to the Editor),** The Hospital, 28 & 29 Southampton Street, Strand, London, W.C.

The Cost of Subscription, post free, is at follows:—Per week, 3½d.; per quarter, 3s. 3d.; per half-year, 6s. 6d.; per annum, 13s. Foreign and Colonial:—Per annum, 19s. 6d., payable, in all cases, in advance.

### BOOKS RECEIVED.

C. Griffin and Co.

"An Introduction to Midwifery." Sixth Edition. By A. Donald, M.D.

A. Siegle.

"Electrical Methods in Affections of the Stomach." By G. Herschell, M.D.

University Tutorial Press.

"Matriculation Directory." XL. June 1905.

Walter Hill.

"The Holidays, 1905."

International Journal of Surgery Company, New York.

"The Surgical Assistant." By W. M. Brickner, B.S.

### Medical Appointments.

**William Johnstone Cameron**, M.B., Ch.B.Edin., Public Vaccinator to Mildura, North-Western District of Victoria, Australia. **Robert Hogarth Clay**, M.D., M.R.C.P.Edin., Honorary Medical Adviser to the Devon and Cornwall Home for Consumption, Didsworthy. **H. W. Collier**, M.B., B.S., Clinical Assistant to the Chelsea Hospital for Women. **W. E. Manderson Corbett**, L.R.C.P.&S.Irel., Honorary Medical Adviser to the Devon and Cornwall Home for Consumption, Didsworthy. **Colin Gray**, M.B., Ch.B.Melb., Public Vaccinator for the Midland District, Victoria, Australia, *pro tem.* **William Waugh Hope**, M.B., Ch.B.Melb., Acting Officer of Health for the Shire of Colac, Victoria, Australia, *pro tem.* **James Herbert Ivey Ingham**, M.B., Ch.B.Melb., Public Vaccinator for the North-Western District and to be Acting Health Officer for the Shire of Dimboola, Victoria, Australia. **George Jackson**, F.R.C.S.Eng., F.R.C.P. Lond., Honorary Medical Adviser to the Devon and Cornwall Home for Consumption, Didsworthy. **Colin Dunrod Lindsey**, M.A., M.D., B.C., D.P.H.Cantab., Honorary Medical Adviser to the Devon and Cornwall Home for Consumption, Didsworthy. **W. B. Maurice**, M.R.C.S., L.R.C.P., Clinical Assistant to the Chelsea Hospital for Women. **W. Robinson**, L.R.C.P., L.R.C.S., Clinical Assistant to the Chelsea Hospital for Women. **William Ambrose Spring**, M.B., Ch.B.Melb., Acting Officer of Health for the Shire of Ballarat, Victoria, Australia, *pro tem.* **Mark Ronald Taylor**, L.R.C.P.Lond., M.R.C.S., Medical Officer of Health of Helston, Cornwall. **Edwin Josiah Toye**, M.D., B.S.Lond., F.R.C.S.Eng., L.R.C.P.Lond., Medical Officer of Health for the Northam (Devon) Urban District Council. **A. J. J. Triado**, M.B., Ch.B.Vict., District Medical Officer of Lawlers, West Australia. **H. Woolmington Webber**, M.D., M.S.Lond., F.R.C.S.Edin., Honorary Medical Adviser to the Devon and Cornwall Home for Consumption, Didsworthy. **Walter Ley Woollcombe**, F.R.C.S.Edin., M.R.C.S., L.R.C.P.Lond., Honorary Medical Adviser to the Devon and Cornwall Home for Consumption, Didsworthy. **Edward Arthur Wright**, M.B., M.S. Edin., Honorary Consulting Surgeon to the Huddersfield Infirmary. **Leonard Youatt**, M.B., B.Ch., D.P.H.Vict., Certifying Surgeon under the Factory and Workshop Act for the Prescot District of the county of Lancaster.

*Published by*
THE SCIENTIFIC PRESS, LTD.
28 & 29 Southampton Street,
Strand, London, W.C.

### New Work for District and Private Nurses.

### SIMPLE SANITATION: THE PRACTICAL APPLICATION OF THE LAWS OF HEALTH TO SMALL DWELLINGS.

Limp cloth, 1/- net, 1/2 post free. By M. LOANE, Superintendent of District Nurses, Portsmouth. Limp cloth, 1/- net, 1/2 post free.

With an Introductory Chapter on Sanitary Legislation by Dr. A. Mearns Fraser, Medical Officer of Health, Portsmouth.

This work should prove of great practical assistance to Private and District Nurses, as it contains Chapters on Sanitary Legislation, Water, Air and Ventilation, Drainage and Disposal of Refuse, Household Management, Personal Hy giene, Disinfectants, &c.

### The Book for the C.M.B.

### A COMPLETE HANDBOOK OF MIDWIFERY.

6/- net, 6/4 post free. By J. K. WATSON, M.D., Author of "A Handbook for Nurses." 143 Illustrations. 6/- net, 6/4 post free.

This book has been compiled in accordance with the requirements of the Central Midwives Board, and will prove not only the best text-book for those entering for the next exam., but is so complete as to be of greatest value to the Qualified Midwife for immediate reference in cases of emergency.

### Useful Text-books for Probationers.

### NURSING: ITS THEORY AND PRACTICE.

Cloth, 3/6 post free. By PERCY G. LEWIS, M.D., M.R.C.S., L.S.A. Cloth, 3/6 post free.

Profusely Illustrated.

A complete text-book of Medical, Surgical, and Monthly Nursing of the utmost value to Probationer Nurses.

### PRACTICAL GUIDE TO SURGICAL BANDAGING AND DRESSINGS.

2/- post free. By WM. JOHNSON SMITH, F.R.C.S. 2/- post free.

A useful guide to Bandaging and Dressing, written especially for Nurses by a surgeon of great experience. The work is up to date and the information thoroughly reliable.

### THE NURSES' PRONOUNCING DICTIONARY OF MEDICAL TERMS AND NURSING TREATMENT.

2/- post free. By HONNOR MORTEN. 2/- post free.

This Dictionary has been used by Nurses for many years, and the fact that it has been recently revised and the phonetic pronunciations added by Miss M. I. Burdett should greatly enhance its value as a work of reference. Its size enables it to be carried comfortably in the apron pocket, which is a strong point in its favour.

*The above works can be obtained of all Booksellers, or of*
The SCIENTIFIC PRESS, Ltd., 28 and 29 Southampton St., Strand, London, W.C.

### The NURSING MIRROR.

**The Nurse's Own Paper.**

**Price ONE PENNY Weekly.**

Of all Booksellers and Newsagents, or posted direct from the Office.

*Write for Subscription Rates to The Manager,*
THE NURSING MIRROR, The Hospital Building, 28 & 29 Southampton Street, Strand, London, W.C.

